# A Methodology for Multihazards Load Combinations of Earthquake and Heavy Trucks for Bridges

**DOI:** 10.1155/2014/126270

**Published:** 2014-05-04

**Authors:** Dezhang Sun, Xu Wang, Baitao Sun

**Affiliations:** ^1^Department of Lifeline Engineering, Institute of Engineering Mechanics, China Earthquake Administration, Harbin 150080, China; ^2^State Key Laboratory Breeding Base of Mountain Bridge and Tunnel Engineering, Chongqing Jiaotong University, Chongqing 400074, China

## Abstract

Issues of load combinations of earthquakes and heavy trucks are important contents in multihazards bridge design. Current *load resistance factor design* (LRFD) *specifications* usually treat extreme hazards alone and have no probabilistic basis in extreme load combinations. Earthquake load and heavy truck load are considered as random processes with respective characteristics, and the maximum combined load is not the simple superimposition of their maximum loads. Traditional Ferry Borges-Castaneda model that considers load lasting duration and occurrence probability well describes random process converting to random variables and load combinations, but this model has strict constraint in time interval selection to obtain precise results. Turkstra's rule considers one load reaching its maximum value in bridge's service life combined with another load with its instantaneous value (or mean value), which looks more rational, but the results are generally unconservative. Therefore, a modified model is presented here considering both advantages of Ferry Borges-Castaneda's model and Turkstra's rule. The modified model is based on conditional probability, which can convert random process to random variables relatively easily and consider the nonmaximum factor in load combinations. Earthquake load and heavy truck load combinations are employed to illustrate the model. Finally, the results of a numerical simulation are used to verify the feasibility and rationality of the model.

## 1. Introduction

The current American bridge design specifications are mainly based on AASHTO load and resistance factor design methodology, which are fully calibrated against gravity load and live load. In AASHTO* LRFD bridge design specifications* [1], a typical bridge is designed for 75 years' service life and load distributions are assumed to be normal and resistance to be lognormal. And a reliability index 3.5 is used to calibrate the safety of the bridges in strength I limit state. However, when extreme loads are considered, the sectional dimensions and construction cost would be increased substantially if similar criteria are placed [2]. Against extreme loads, each one has its own unique approach, principally because the data and statistics are rare. This fact makes it difficult to properly consider extreme loads in a consistent fashion.

In recent years, bridges are subject to more and more diversified natural hazards, and the frequencies of various extreme hazards also seem to be increasing, which bring great threats to safety of the bridges so as to bring potential danger to personal life and property safety. The concept of multihazards design is quoted in many papers and reports at present [3–7]. An organization of Multidisciplinary Center for Extreme Events Research (MCEER) was established, and one of the tasks is to develop a framework to systematically expand the LRFD specification to multihazard (MH)-LRFD for bridges. Many scholars and organizations have made many studies on the effect of a single extreme load on bridge [8–11]; however, there are only a few reports of studies on the effect of multiple extreme loads on performance of the bridge during its whole service life [12–14].

When there is only one extreme load (such as earthquakes, scour, vessel collisions, etc.), the load combination includes an extreme load and dead load and in such case no matter whether the extreme load is a random variable or random process, its combination only relates to the maximum value. It is quite a different situation when two or more extreme loads are considered in the combinations. Because the intensity of extreme load is a function related to time and even two extreme loads occur simultaneously in a short time, the probability for them to reach their highest intensities at the same time is very low, which means that when multiple extreme loads are combined, the maximum combined load is not simply the superimposition of their maximum value. Among many load combination models, Ferry Borges-Castaneda model [15], Turkstra's rule [16], and Wen's method [17, 18] are the most frequently used. However, they have their own advantages and disadvantages. Loads lasting duration and occurrence probability are considered in Ferry Borges-Castaneda model, and the conversion of random process to random variables and the combination with other random variables are well described; however, it has strict requirement for time interval. Turkstra's rule considers that a load reaches the maximum value during design life of the bridge combined with the instantaneous value (or mean value) of another load. This rule is more practical and reasonable, but the results are generally unconservative [19]. Wen's load coincidence method does not give a rational explanation of corelation in the probability of failure of different events.

In this paper, a model based on conditional probability is proposed to combine advantages of the Ferry Borges-Castaneda model and Turkstra's rule to achieve a more reasonable result of load combination. Then earthquake load and heavy truck load combinations are employed to illustrate the model.

## 2. Basic Modeling

**Figure 1:**
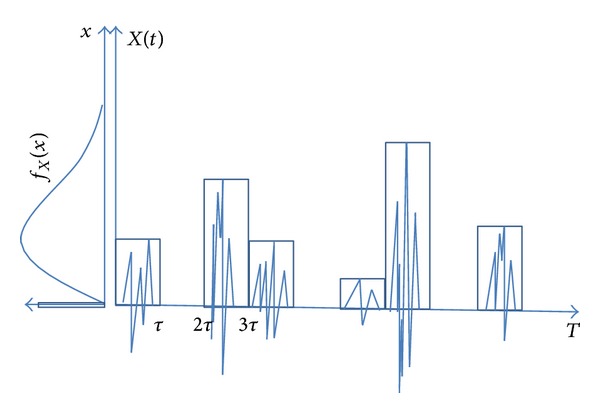
Assumption of load in its duration.

**Figure 2:**
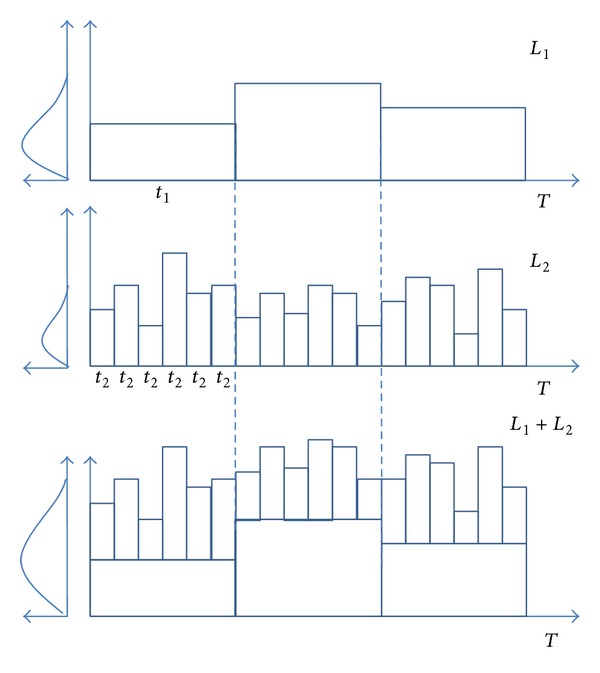
Model of Ferry Borges-Castaneda method.

Earthquake load usually lasts for a short time and has enormous destructive power, while the frequency of heavy trucks is relatively high and some overloaded trucks or multiple trucks “running side by side” bring damage to the bridge greatly. It should be noticed that the maximum 75-year truck load was usually great and can be considered as extreme load. Generally, extreme load is of low occurrence probability and great intensity and even lower chance of simultaneous occurrences. Even the extreme loads do occur simultaneously; the chances that they achieve their respective maximum value are very low. Therefore, the common occurrence frequencies of different extreme loads are generally not considered [20]. According to Ferry Borges-Castaneda model, Turkstra's rule, and Wen's method, it is assumed that the earthquake load and heavy truck load comply with stable Poisson process; the probability and times at any point of time during design service life of the bridge comply with Poisson distribution. The load histories of earthquake load and heavy truck load during their respective time intervals are the functions of time and intensity accompanied with great randomness, which makes load combination very difficult. To simplify the combination, it is assumed that the earthquake and heavy truck loads maintain the maximum value during their respective time history (as shown in Figure 1). Although such assumption is conservative, the problem of combination is greatly simplified. To a certain extent, “combination of time history” is not necessarily more advantageous than the simplified combination because bridge design also takes the maximum combination into consideration and it also increases the level of calculation difficulty. Ferry Borges-Castaneda model that considers load lasting duration and occurring probability well describes random process converting to random variables and load combinations of earthquake and heavy truck load. The basic combination thought of Ferry Borges-Castaneda model can be expressed as Figure 2.

Note that in Figure 1 *f*_*X*_(*x*) is the probability density of load in *τ* interval, which is the function of time and load intensity. The occurrence of load is determined through Poisson distribution. When only two random processes are considered, assume that the time interval of load 1 is *t*_1_, that of load 2 is *t*_2_, and *t*_1_ ≥ *t*_2_. Under only one kind of loads, the maximum value of load 2 in *t*_1_ interval can be described with (1) and the maximum value of load 1 in *T* period can be obtained through

(1) $$\begin{array}{c} x_{\max,t_{1}}=\underset{t_{1}}{\max}\left[x_{2}\right], \end{array}$$

(2) $$\begin{array}{c} x_{\max,T}=\underset{T}{\max}\left[x_{1}\right]. \end{array}$$

One of the main tasks of load combination is to find the maximum value of combined load under multiple kinds of loads in the service life of bridge. Therefore, based on the assumptions of load definition of Ferry Borges-Castaneda model, the maximum value of load combination in *t*_1_ interval is max⁡  (*x*_max⁡,*t*_1__ + *x*_1_) (see Figure 2); then the maximum value [21] in *T* period can be obtained through

(3) $$\begin{array}{c} F_{X\;\max,T}\left(x\right)={\left[F_{X}\left(x\right)\right]}^{n}, \end{array}$$

where *F*_*X* max⁡,*T*_(*x*) is the cumulative distribution function (CDF) of the maximum value of combined load in *T* period; *F*_*X*_(*x*) is the cumulative distribution function of load combination in *t*_1_ interval; *n* is integer, which can be obtained from *T* and *t*; namely, *n* = *T*/*t*.

## 3. A Modified Model for Load Combination

According to the Ferry Borges-Castaneda model, the service life of the bridge can be divided into equal time interval *t*; if it is small enough, the results are nearly precise. However, the interval of time depends on many factors; it is not small enough and the duration of the maximum load effect of each load within a time interval, such as heavy truck passing a common bridge, is relatively certain, while this “duration” is usually shorter than time interval *t*. Therefore, the probability of simultaneous occurrences is not equal to the product of respective occurrence probabilities of the loads. If they are made to be equal, the probability of maximum load effect is exaggerated so as to lead to conservative results. It is assumed that the probability of occurrence probability of load 1 within interval *t* is *p*_1_, that of load 2 is *p*_2_, and the probability of simultaneous occurrences is *p*. The probability of load 1 event in its duration is *p*_1*r*_ and the probability of load 2 event in its duration is *p*_2*r*_; then we can find

(4) $$\begin{array}{c} p_{1}\cdot p_{2}\neq p_{1r}\cdot p_{2r};\;p\geq p_{1r}\cdot p_{2r}. \end{array}$$

Besides, power conversion of probability curves is required when using Ferry Borges-Castaneda model. For example, converting probability curve in a long time *T*_0_ to that in an interval *t*, when *T*_0_ ≫ *t*, the probability curves may have error after root of large number and the error is cumulative, especially for curves with values on negative part of *X* axis (such as normal distribution curve). For earthquake, annual probability curve is obtained from the USGS Mapping [22] and the computation of multiple powers for curve conversion is needed. Therefore, the number of powers should be controlled to prevent loss of data because the number is too large.

Based on these problems, Ghosn et al. [20] improved the Ferry Borges-Castaneda model. However, Ghosn believed that there must be trucks on the bridge within the duration of earthquake. This assumption is one of the important reasons causing the conservative results of Ghosn's model. According to the report of Nowak and Szerszen [23–26], it is assumed that it takes 8 seconds for a heavy truck to pass through a common bridge and 600 trucks to pass through the bridge per day, and seismic excitation duration is 40 seconds. Also it is assumed that the heavy truck passing through a bridge during the earthquake meets the condition of Poisson process. The effective lasting duration of earthquake is *τ*_*e*_ and the time for the heavy truck passing through the bridge is *τ*_*t*_; the probability of the heavy truck on the bridge during the earthquake is *P*(*e*_*e*,tr⁡_); then,

(5) $$\begin{array}{c} P\left(e_{e,\mathrm{tr}}\right)\leq 1-e^{-\lambda (\tau_{e}+\tau_{t})}, \end{array}$$

where *λ* is the frequency of heavy trucks.

The probability is less than 30% when (5) holds the equal mark. Since the times of trucks passing through a common bridge are limited in a given exposure time, the probability has little change when 6 and more trucks passing through the bridge based on Poisson distribution.

Since the number of earthquakes in a 75-year return period is usually few, it is mentioned in the report of Ghosn et al. [20] that, according to USGS Mapping, the number of earthquake in San Francisco in a 75-year return period will be 600 and 150 in Seattle, 38 in Memphis, 30 in New York, and 1 in St. Paul, which are far less than millions of heavy trucks in bridge's service life.

**Figure 3:**
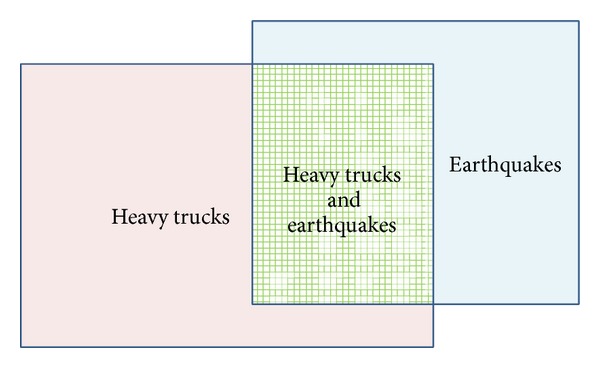
Illustration of heavy trucks and earthquakes sample space.

The earthquake and heavy truck load combinations are different from other extreme load combinations (except for scour), and the probability of simultaneous occurrences cannot be ignored just as described above. In case of earthquake load and heavy truck load combination, the combination situations include heavy truck load only, heavy truck load and earthquake load, and earthquake load only (as shown in Figure 3). If they are put together (viz., they are treated in one sample space), the sampling probability of earthquake is small since the number of trucks is far more than that of earthquake; thus the most concerned probability of earthquake effect is “very small,” which is obviously abnormal. Earthquake and heavy truck are two kinds of loads and their impact levels are different. In other words, although the number of earthquake occurrence is less, its destructive power and impact are larger. Besides, from a different point of view, the probability of earthquakes to come across heavy trucks is obviously different from that of heavy trucks to come across earthquakes while passing the bridge. Since there are many heavy trucks, the probability of heavy trucks to come across earthquake while passing the bridge is far less than that of the earthquake to come across heavy trucks. For a common bridge, the time interval for heavy trucks to cause maximum load effect is shorter than the lasting duration of earthquake. In such case, if heavy trucks are treated as the benchmark, no uniform and definite results will be obtained (because multiple heavy trucks may correspond to one earthquake).

To sum up, heavy trucks and earthquakes load are two kinds of loads with different natures and should be put in different sample spaces for their combination. The destructive power of earthquake is larger and the lasting duration of earthquake is longer than that of heavy trucks passing a common bridge. Therefore, it is proper to consider earthquake as the condition for load combination.

**Figure 4:**
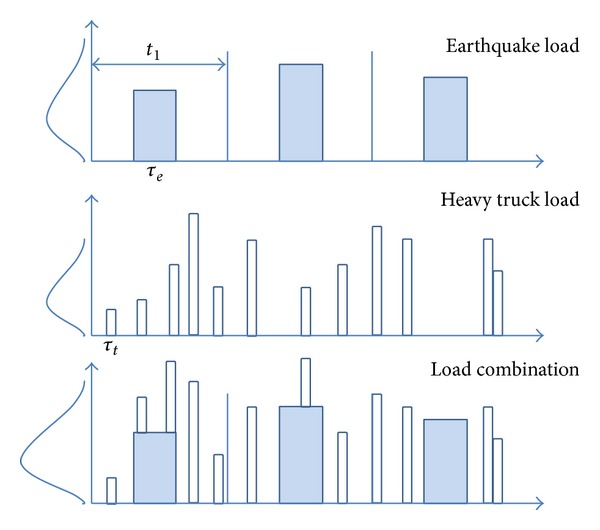
Illustration of load combination of modified model, where *τ*_*e*_ and *τ*_*t*_ are the event durations of earthquake and heavy truck, respectively.

In consideration of the complexity of earthquake and heavy truck load combinations, a modified model will “investigate and review” heavy trucks based on earthquake; namely, there may be or may not be heavy trucks on the bridge during the effective time interval of earthquake. If former condition happens, there may be multiple trucks at the same time or only one truck, as shown in Figure 4. It is assumed that the load effect within each time *t*_1_ is independent and has the same distribution.

Analysis of the truck issues based on earthquake condition will make the most concerned extreme load such as earthquake load outstanding and clear, make load effect combination clarified, and prevent the “confusion” when sample spaces are mixed, so as to simplify the concept of load effect combination. During each time *t*_1_ (a long time period), incorporating the conception of Turkstra's rule, the difference is that the heavy truck load here is not instantaneous value or mean value. Since heavy truck is “observed” when earthquake has happened, earthquake should be analyzed by number and then earthquake and heavy truck combination could be considered. The expected numbers of earthquakes are provided by USGS Mapping Project.

The cumulative distribution, *F*_*e*_(*x*), of load for one earthquake's occurrence is defined by

(6) $$\begin{array}{c} F_{e}\left(x\right)={\left[P_{y}\left(x\right)\right]}^{1/n_{ey}}, \end{array}$$

where *n*_*ey*_ is the annual average number of earthquake;  *P*_*y*_(*x*) is the annual cumulative probability function curve of earthquake load.

Note that *n*_*ey*_ may not always appear as an integer for calculation accuracy and convenience according to different areas and is related to total occurrence number of earthquake.

On the basis of reliability theory, combining heavy truck with earthquake, the cumulative distribution function of possible maximum truck load *F*_tr⁡_(*x*) is calculated using the following equation:

(7) $$\begin{array}{c} F_{\mathrm{tr}}\left(x\right)={\left[F\left(x\right)\right]}^{\left(n_{\mathrm{tr}}\cdot n_{e}\right)}, \end{array}$$

where *n*_tr⁡_ is the quantity of heavy trucks in effective combined action time of earthquake and heavy trucks; *F*(*x*) is the load cumulative distribution function of a single heavy truck; *n*_*e*_ is the number of earthquake meeting with heavy trucks.

The maximum value of combination of earthquake load and heavy truck load is obtained through (3). The probability of the earthquakes “alone” (without a heavy truck passing by) is considered through the probability of earthquake occurrence *P*(*e*_only_). *P*(*e*_*e*,tr⁡_) is the probability of combined action of earthquake and heavy truck; then there is *P*(*e*_*e*,tr⁡_) + *P*(*e*_only_) = 1. Based on earthquake condition, the modified model can be expressed as

(8) P(X≤x,e)=P(X≤x,ee,tr⁡)+P(X≤x,ee,only)=P(X≤x ∣ ee,tr⁡)·P(ee,tr⁡) +P(X≤x ∣ ee,only)·P(eonly)=Fe,tr⁡(x)·P(ee,tr⁡)+Fe,only(x)·P(eonly),

where *F*_*e*,tr⁡_(*x*) is the cumulative distribution function of load combining earthquake with heavy trucks; *F*_*e*,only_(*x*) is the cumulative distribution function of load of the rest earthquakes “alone.”

Therefore, considering both combination of earthquakes and heavy trucks and earthquakes “alone,” the probability density function of total load is calculated as

(9) $$\begin{array}{c} f_{\text{com}}\left(x\right)=P\left(e_{e,\mathrm{tr}}\right)f_{e,\mathrm{tr}}\left(x\right)+P\left(e_{\text{only}}\right)f_{e,\text{only}}\left(x\right), \end{array}$$

where  *f*_*e*,tr⁡_(*x*) is the probability density function of load combining earthquakes with heavy trucks; *f*_*e*,only_(*x*) is the probability density function of load of earthquakes “alone.”

Since the methodology in this paper is based on the condition of earthquake, the whole load combination is divided into those with earthquake condition and without earthquake condition, which are “alternative.” The “circumstance” without earthquake is the situation of remaining heavy trucks, which refer to those not coming across earthquake (since the number of trucks coming across earthquake is limited and is ignorable compared to millions of trucks; it is assumed that the number of “remaining” heavy trucks remains the same). Therefore, the joint cumulative probability distribution function of earthquake and heavy truck load combination is given as

(10) $$\begin{array}{c} F_{\max,T}\left(x\right)=F_{\text{com}}\left(x\right)\cdot F_{\mathrm{tr},\text{only}}\left(x\right), \end{array}$$

where *F*_max⁡,*T*_(*x*) is the joint cumulative probability function of earthquake load and heavy truck load in time *T* period; *F*_com_(*x*) is the cumulative probability function in earthquake condition; *F*_tr⁡,only_(*x*) is the cumulative probability function of maximum value of heavy trucks in time *T* period.

To sum up, the procedures of load (effects) combination based on earthquake condition are generally summarized as follows.

1. Determine the time intervals of earthquake load and heavy truck load.
2. Calculate the occurrence frequency *λ* of heavy truck load during the time interval according to the stable Poisson process assumption and calculate the occurrence probability and probability distribution function of heavy truck load within the interval.
3. Check the expected (or annual) number of earthquakes within *T* period with reference to USGS Mapping and calculate the distribution of maximum earthquake load within *t*_1_ using (6).
4. Determine the probability of earthquake and heavy trucks coming across each other.
5. Determine the number of heavy trucks of occurrence within the time interval of earthquake and calculate the possible maximum value distribution function with (7) when the heavy trucks and earthquake are combined.
6. Determine the number of earthquakes combined with the trucks and calculate the cumulative distribution function of the combined portion of earthquake and heavy trucks.
7. Using (8) and (9), calculate the maximum value distribution function of combined portion of earthquake and heavy trucks and the portion of earthquake alone under earthquake condition.
8. Calculate the final maximum value distribution using (10).
9. If constant load effect such as dead load participates in the combination with other load effects, it should be combined with the dead load effect within *T* (such as 75 years) period.

## 4. Numerical Simulation and Example

**Figure 5:**
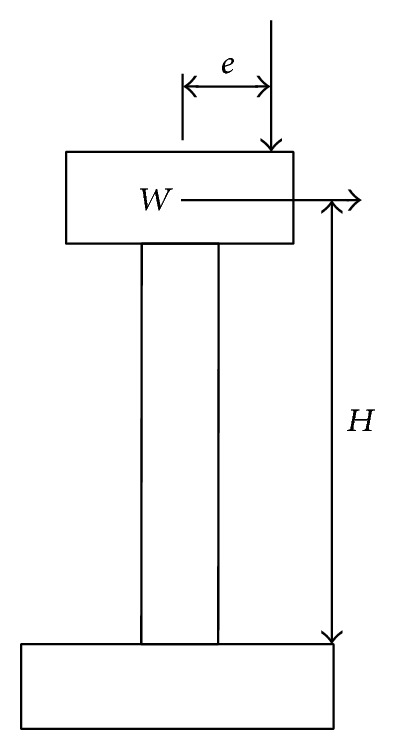
Computing model of the bridge.

In order to illustrate the feasibility and clarity of the methodology of load combination described in the preceding section, a simple example of load combination simulation is presented here. In order to illustrate the method clearly, some simplifications are made, which include the assumption that the earthquake load is given by *M* = *W* · *A* · *H*, where *M* is the column bent moment, *W* is superstructure weight, *A* is peak ground acceleration (PGA), and *H* is column calculation height. The truck load is given by *M* = *F* · *e*, where *F* is the truck weight and *e* is the eccentricity between the vertical center axis of the truck and the vertical axis of the column. The effects of soil and secondary effect of gravity are ignored. The computing model of the bridge is shown in Figure 5. The main configuration of the bridge is *W* = 500 ton, *H* = 6.0 m, and *e* = 4 m, and the bridge locates in Seattle, USA. Further it is assumed that the maximum number of trucks on one lane is two in one direction and in a special site heavy truck may have an average number of 1000. Moses [27]suggested heavy trucks approach a normal distribution with a mean of 300 kN and a standard deviation of 80 kN (coefficient of variable: COV = 26.5%) and more heavier truck situations can be obtained through basic assumption above. The earthquake curve can be found from USGS mapping, which includes PGA and frequency of exceedance and could be converted to cumulative curve [13, 14]. According to USGS mapping, the expected number of earthquakes in Seattle is 2, and then there will be 150 in 75 years' service life of common bridge. The results are given in Figure 5 to Figure 14.

**Figure 6:**
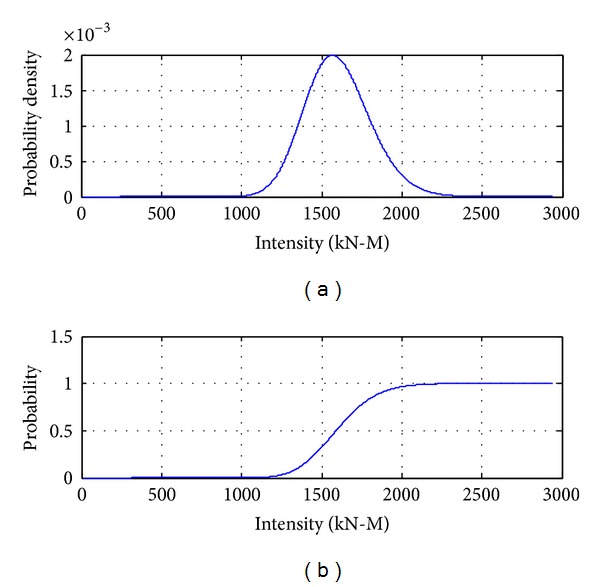
The possible probability distribution of trucks combined with earthquake.

**Figure 7:**
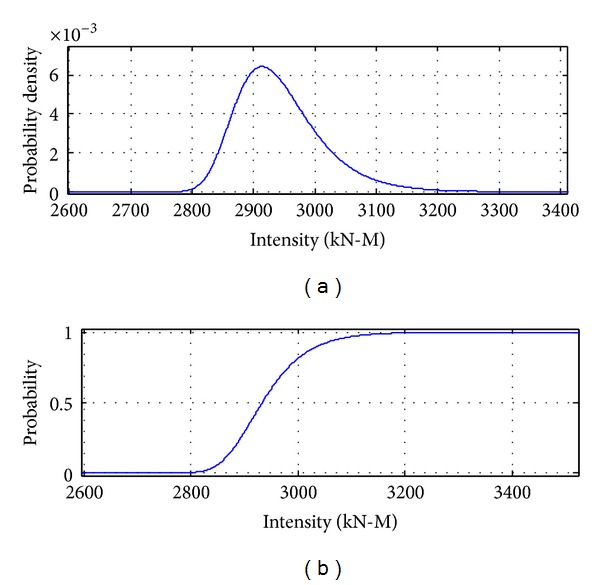
Probability distribution of truck load effects with maximum number.

According to Figures 6 and 7, the maximum truck load combined with earthquake is between the single common truck load and the maximum 75-year truck load, which is reasonable because the larger the earthquake is, the smaller the occurrence probability is and the smaller the probability of coming across heavier trucks will be, and, on the contrary, the smaller the earthquake is, the larger the occurrence probability is and the larger the probability of coming across heavier trucks will be.

**Figure 8:**
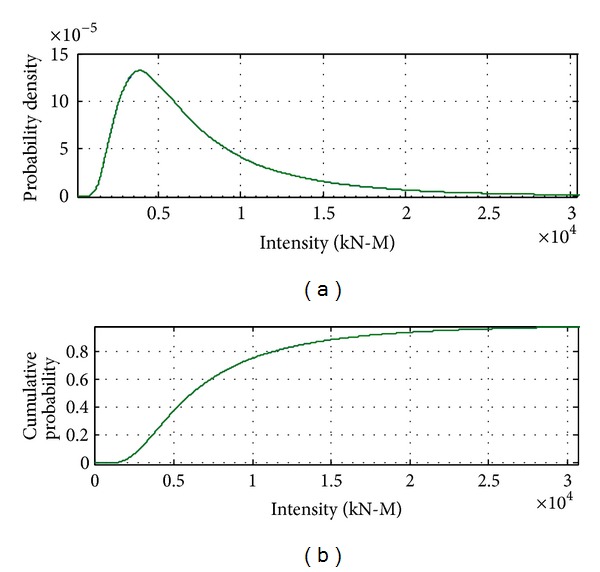
Probability distribution of earthquake load effects in 75 years.

**Figure 9:**
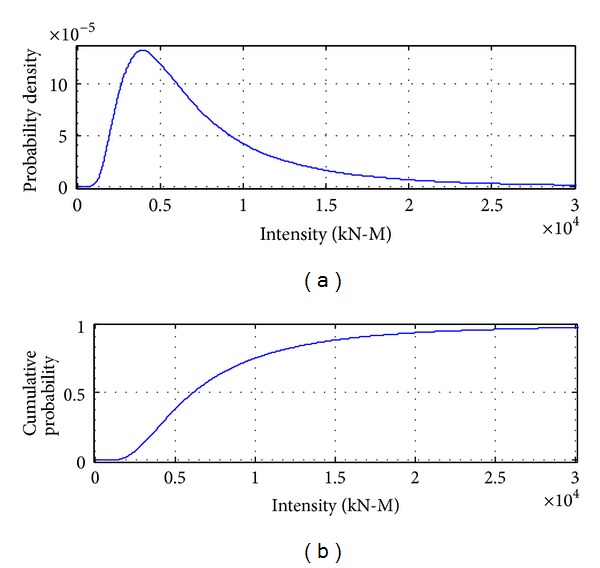
Probability distribution of earthquake load effects alone.

**Figure 10:**
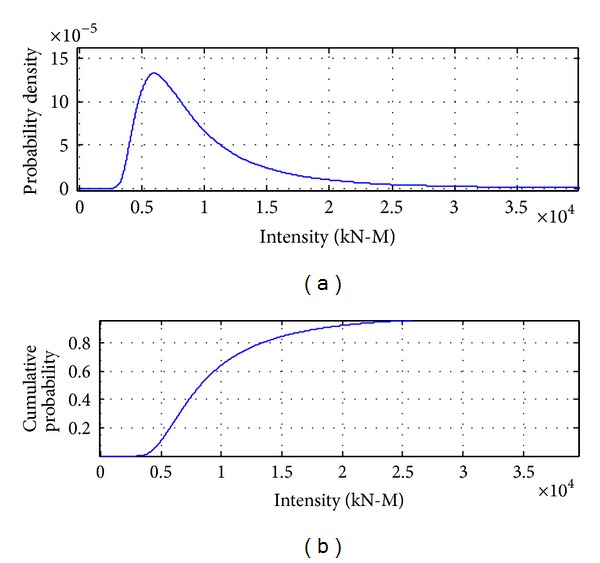
Probability distribution of earthquake load effects combined with truck load effects.

**Figure 11:**
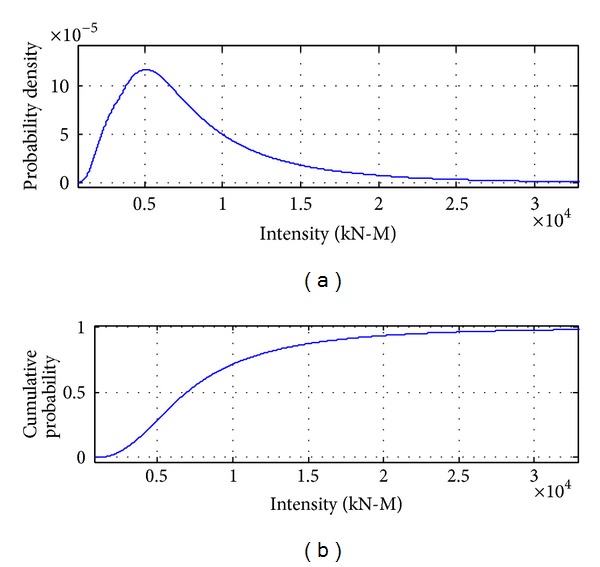
Probability distribution of earthquake load effects alone combined with those of earthquake and truck load effects combination portion.

**Figure 12:**
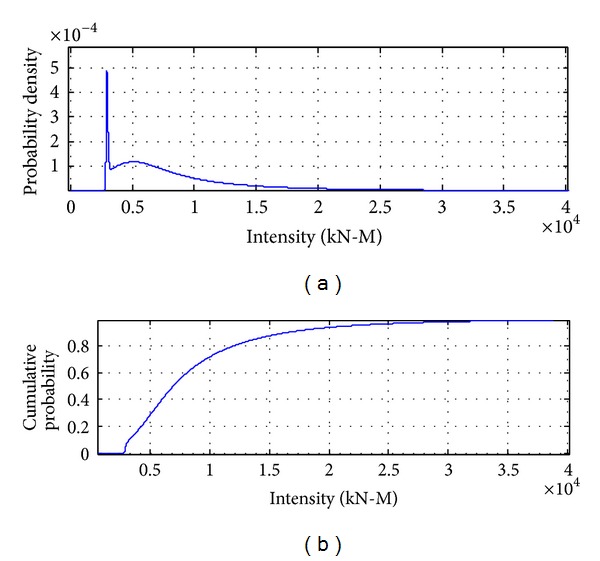
Probability distribution of load effects under earthquake condition combined with remaining truck load effects in 75 years.

Figure 8 to Figure 12 show the results of combinations under earthquake condition. According to the results in Figures 9 and 10, the curves of load combinations change after the situation of earthquake coming across trucks is considered. In this example, because the probability of coming across between heavy trucks and earthquake is relatively small, we can see from the results that the shape of probability curve under earthquake only is similar with that of 75 years' maximum earthquake load curve, but with the mean moving leftwards.  Figure 11 shows the load combination of earthquake load effects alone combined with those of earthquake and truck load effects combination portion, and the result is of similar shape with the maximum earthquake load in 75 years, but with the mode larger than that under earthquake only. Figure 12 shows the combination of the maximum load under earthquake condition and the maximum load of “remaining” heavy trucks. Consideration of the comparison between them is the solution of their different sample spaces, and the one with smaller contribution according to the comparison will be omitted.

**Figure 13:**
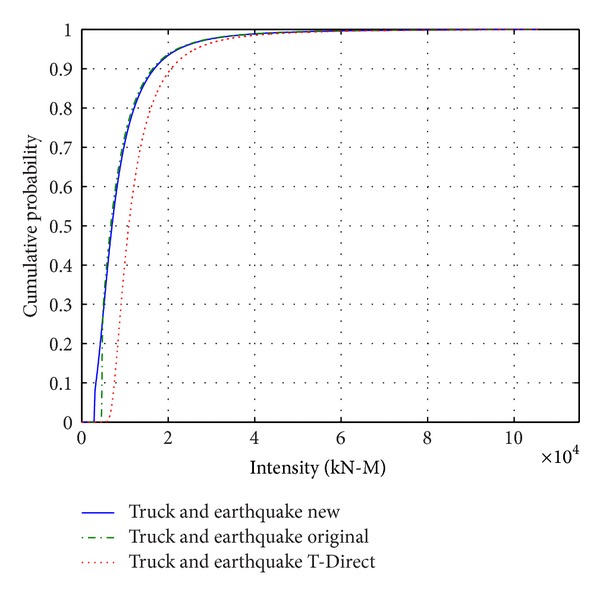
Comparison of cumulative probability curves with different methods.

**Figure 14:**
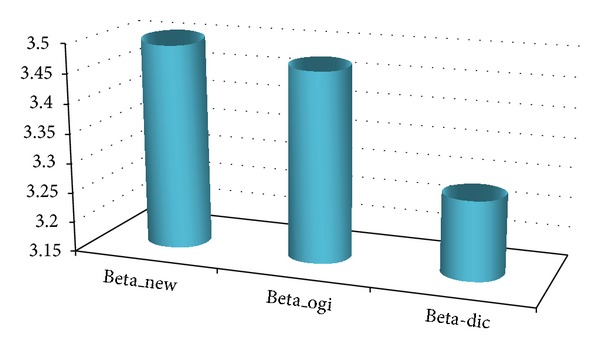
Comparison of reliability index corresponding to the curves in Figure 13.

Figure 13 shows general comparison of the results using the modified model with the results of the traditional Ferry Borges model. In the figure, “Truck and earthquake new” is the cumulative probability curve of maximum value calculated with the model in this paper, “Truck and earthquake original” is the probability curve calculated with the traditional model (with reasonable time interval), and “Truck and earthquakeT-Direct” is the probability curve of direct load combination of the earthquake and heavy truck loads in their 75 years. According to the results, we can observe that (1) “Truck and earthquakeT-Direct” curve is on the right end; “Truck and earthquake original” curve and “Truck and earthquake new” curve are partially crossing and lapping over and are close. (2) According to the value, their means are truck and earthquakeT-Direct > truck and earthquake original > truck and earthquake new and the coefficients of variation are close. Figure 14 shows the comparison on the reliability index corresponding to Figure 13. According to the bar graph, the reliability index of the new model is the maximum that is nearest to the 3.5, followed by the original model, and that of the direct load combination in 75 years is the minimum.

## 5. Conclusions

According to the disadvantages of Ferry Borges-Castaneda model, the unreasonable assumption of Ghosn's improved model, and the merit of Turkstra's rule, a modified model of multihazard load combination is proposed in this paper. The model considers load effect combinations based on earthquake conditions.

Compared with the traditional Ferry Borges-Castaneda method, the main advantages of this methodology include the following: it can prevent the complexity of sampling in multiple load sample spaces and it pays more attention to extreme load such as earthquake load. Based on load occurrence frequency, this methodology does not focus on one extreme load effect but on their joint effects. This methodology combines advantages of Ferry Borges-Castaneda model and idea of Turkstra's rule. It is like the Ferry Borges-Castaneda model in general and the Turkstra's in partial, whose implications are more definite and the thought is clear.

Another advantage is to prevent the cumulative error due to the root of large numbers of earthquake curve. Since the Ferry Borges-Castaneda model requires earthquake curve to be reduced within time interval *τ* (for e.g., 30 seconds), cumulative distribution of earthquake curve generally requires the root of 1051200 (year to time interval *τ*), while the new method generally needs only root of a small number. Besides, the product of multiple cumulative distributions is used to calculate the maximum value of load combination in this model, which will simplify multiple load combination greatly. If the probability of multiple extreme loads simultaneous occurrences is very low, their combination is the product of their respective cumulative distribution, which simplifies the calculation a lot.

With respect to the simulation value, the means of the new method is the minimum and the maximum with reliability index, while the value of combination in *T* period is the most conservative. Furthermore, the results of different regions may be different because the earthquakes of the chosen regions are greatly different. For example, earthquakes in California are frequent, and thus the load combination is greatly affected by earthquake. Earthquakes in St. Paul are less frequent, and thus the impact is mainly from the trucks. For New York, the impacts from neither earthquake nor trucks can be ignored and the participation of earthquake and heavy trucks in the results is approximate.
